# *Evodia rutaecarpa* and Three Major Alkaloids Abrogate Influenza A Virus (H1N1)-Induced Chemokines Production and Cell Migration

**DOI:** 10.1093/ecam/nep238

**Published:** 2011-06-08

**Authors:** Wen-Fei Chiou, Han-Chieh Ko, Bai-Luh Wei

**Affiliations:** ^1^National Research Institute of Chinese Medicine, Taipei 112, Taiwan; ^2^Institute of Life Science, National Taitung University, Taitung, Taiwan

## Abstract

*Evodia rutaecarpa* is commonly used as an anti-inflammatory herbal remedy in traditional Chinese medicine. In this study, the ethanol extract of *E. rutaecarpa* (ER) and three major quinazoline alkaloids dehydroevodiamine (DeHE), evodiamine (Evo) and rutaecarpine (Rut), isolated from ER were employed to study their inhibitory effects against influenza A virus (H1N1)-induced chemokines production in A549 lung epithelial cells as well as on chemokines-evoked cell recruitment in HL-60-differentiated macrophages. The results showed that ER was a potent inhibitor of RANTES secretion by H1N1-inoculated A549 cells (IC_50_: 1.9 ± 0.4 *μ*g ml^−1^). Three alkaloids, although to differing extents, all concentration dependent, inhibited H1N1-induced RANTES production with Evo consistently being the most potent among these active components. ER also moderately and significantly inhibited H1N1-stimulated MCP-1 production in A549 cells. This was mimicked by Evo and Rut, but not DeHE. In the macrophage recruitment assay, both RANTES and MCP-1 markedly evoked cell migration and this phenomenon was significantly suppressed by ER. Evo and Rut, but not DeHE, also had the ability to inhibit cell migration toward RANTES and MCP-1, respectively. In summary, three major alkaloids displayed different potentials for inhibiting chemokines secretion and subsequently cell migration, which could partially explain the activity of ER. As an effective agent to suppress H1N1-induced chemokines production and block chemokine-attracted leukocytes recruitment, *E. rutaecarpa* and its active components may be useful in influenza virus infection-related inflammatory disorders.

## 1. Introduction

An outbreak of infections with a new influenza A (H1N1) virus that was first detected in the USA and Mexico is currently ongoing worldwide. The most important feature of novel influenza A (H1N1) or avian flu (H5N1) immunopathogenesis is the appearance of hypercytokinemia (cytokine storm) that is characterized by the extreme (exaggerated) production and secretion of large numbers and excessive levels of pro-inflammatory cytokines/chemokines. This phenomenon is blamed on the emergence of lethal clinical symptoms, such as extensive pulmonary edema, acute bronchopneumoniae, alveolar hemorrhage, reactive hemophagocytosis and acute respiratory distress syndrome, associated with necrosis and tissue destruction. Many *in vitro*, *in vivo* and clinical studies have pointed out that novel influenza A/H5N1 viruses are very strong inducers of various cytokines and chemokines in both humans and animals [1], for example, RANTES (regulated on activation, normal T cell expressed and secreted) is a potent chemoattractant for monocytes/macrophages, basophils and T cells and has been found in the nasal secretions of patients suffering from upper respiratory tract infection with influenza virus, parainfluenza virus and adenovirus [2, 3]. Macrophage chemotactic protein-1 (MCP-1), a member of the CC chemokines subfamily recruiting and activating mainly monocyte/macrophages in inflammatory sites, is also reported to play a crucial role in the progression of chronic inflammation and multiple sclerosis after viral infection [4, 5]. These chemokines are rapidly induced following infection, and they bind to their receptors to initiate immune cell migration and infiltration. Precisely regulating chemokine expression and subsequent cellular responses likely optimize protection and minimize the deleterious effects of uncontrolled viral invasion.

Traditional Chinese medicine has long been used as a remedy against infectious diseases in China. For example, “Wu-Chu-Yu,” the unripe fruit from *Evodia rutaecarpa*, has long been utilized in traditional Chinese medicine for treating pathogen infections (e.g.; pneumonia bacteria) and inflammation-related disorders such as eczema, ulcerative stomatitis and etcetera [6]. Several alkaloids with biological activity have been identified in *E. rutaecarpa* including three major alkaloids: dehydroevodiamine (DeHE), evodiamine (Evo) and rutaecarpine (Rut) [7]. Pharmacological investigations have revealed different extracts of *E. rutaecarpa*, and its chemical constituents display many biological activities related to inflammation, for example, antinociception, anti-inflammation, immune modulation, nitric oxide (NO) inhibition [8], protection against endotoxin shock in rats and anti-inflammatory activity in human skin [9–14]. However, little is known about their potential against viral infection-mediated chemokine secretion and cell infiltration.

In this study, we attempted to set up two inflammation-related cellular models including (i) activation of human lung epithelial cells by influenza virus-mediated induction of chemokines (RANTES and MCP-1) and (ii) cell migration in response to chemokins in HL-60-differentiated macrophage, which is a crucial determinant of leukocyte trafficking, as two parameters for evaluating the anti-inflammatory potential of the ethanol extract of *E. rutaecarpa* (ER) and simultaneously correlating its anti-inflammatory activities with three major alkaloids (DeHE, Evo and Rut). We found that *E. rutaecarpa* and its active components especially Evo and Rut, may be useful in viral (influenza A) infection-related inflammatory disorders by limiting chemokine secretion and the early phases of macrophage infiltration.

## 2. Methods

### 2.1. Extract Preparation and Alkaloids Isolation

Dried *E. rutaecarpa* (Juss.) Benth was purchased from a local drug store in Taipei and identified by Mr C. J. Chou (Fellow in Pharmacognosy, Principal Investigator in National Research Institute of Chinese). ER was prepared as described in our previous reports [14]. After being vacuum dried, the ER was re-dissolved in dimethyl sulfoxide (DMSO) as a 10 mg ml^−1^ stock solution. Three major components, DeHE, Evo and Rut, were purified from the ER [15, 16]. Their identities were confirmed by comparing their NMR and IR spectra with those reported in the literature [17]. These drugs were all dissolved in DMSO as stock solutions of 10 mM. The final concentration of DMSO in the reaction buffer was <0.25%, and at that concentration, it showed no significant cytotoxicity or biological activity as compared with drug-free samples as reported in our previous report. 

### 2.2. Cell Culture, Virus Preparation and Infection Protocol

Adherent Madin-Darby canine kidney II (MDCK-II) purchased from Food Industry Research and Development Institute (Hsinchu, Taiwan) cells were grown in DMEM supplemented with 10% heat-inactivated, virus- and mycoplasma-free fetal calf serum (FCS), 100 U ml^−1^ of penicillin, 100 *μ*g ml^−1^ of streptomycin and 2 mM l-glutamine (Biological Industries, Israel). Human alveolar epithelial cells (A549) (Industry Research and Development Institute, Hsinchu, Taiwan) were grown in the RPMI 1640 medium (Gibco BRL, USA) supplemented with 10% heat-inactivated FCS, 100 U ml^−1^ of penicillin, 100 *μ*g ml^−1^ of streptomycin, 2 mM l-glutamine and non-essential amino acid. Influenza A virus strain A/PR/8/34 (H1N1) was kindly donated by Dr C. H. Chan, Department of Microbiology and Immunology, School of Medicine, Chung Shan Medical University, Taichung. The virus was propagated in MDCK cells in serum-free DMEM containing porcine trypsin (Sigma, USA) to facilitate infection of cells at 37°C. Virus production was followed by titration of viral hemagglutinin (HA) [18]. Virus seed was stored at aliquots of 10 ml at −70°C.

For the assay, A549 cells were grew in 24-well plastic tissue culture plates (Costar, USA). Confluent monolayers of A549 cells were inoculated with H1N1 at a multiplicity of infection (MOI) of 1 plaque forming unit (PFU)/cell in the serum-free RPMI 1640 medium at 37°C. After a 1-h absorption period, the virus-containing medium was removed, washed with PBS and furthermore incubated in medium containing 1% FCS for 72 h in the absence or presence of various concentrations of ER or three major alkaloids, respectively. Supernatants were collected at 72 h and assayed for human RANTES and MCP-1 [19]. The A549 monolayers in the culture plate were separated from the medium, washed with PBS to remove the dead cells resulting from herbal extracts treatment and the cell viability was determined by staining with MTT (see below).

### 2.3. Cell Viability

Cell respiration, an indicator of cell viability, was determined by the mitochondrial-dependent reduction of 3-(3,4-dimehyl-thiazol-2-yl)-2,5-diphenyl-tetrazolium bromide (MTT) to formazan. The extent of the reduction of MTT to formazan within the  cells was quantified by measuring the optical density at 550 nm.

### 2.4. Chemokines Measurement

Cultured supernatants collected from uninfected and infected A549 cells, or before and after tested agent treatment, were assayed for RANTES and MCP-1 using commercially available ELISA kits according to the procedures provided by the manufacture (Chemicon). All samples were determined in triplicate. The standard concentrations range for RANTES were 0, 31.2, 62.5, 125, 250, 500 and 1000 pg ml^−1^, for MCP-1 were 0, 15.6, 31.2, 62.5, 125, 250, 500 and 1000 pg ml^−1^, respectively.

### 2.5. Culture and Differentiation of HL-60 Cells

Human promyelocytic HL-60 cells (ATCC) were grown in the RPMI 1640 medium supplemented with 10% FCS, 0.1 mM nonessential amino acids, 100 U ml^−1^ penicillin and 100 *μ*g ml^−1^ streptomycin at 37°C under a humidified 95%/5% (v/v) mixture of air and CO_2_. For induced cells to undergo differentiation along the monocyte/macrophage lineage, the cells were seeded onto dishes at 5 × 10^5^ cells ml^−1^ and treated with PMA at doses of 2 nM. After 48 h of treatment, ~>80% of the cells adhere to the dish. The adherent cells were harvested using trypsin/EDTA and used to assess the chemotactic migration.

### 2.6. Chemotactic Migration

Cell migration was assessed using a 24-well chemotaxis chamber with a membrane pore size of 5 *μ*m (Transwell, Corning Costar). Ninety microliters of cell suspension (2 × 10^7^ cells ml^−1^) was added to each of the upper wells in the presence of 10 *μ*l PBS or various concentrations of ER or alkaloids for 30 min. Recombinant RANTES and MCP-1 proteins (R&D, USA) were added to the lower well of the chamber to assess chemoattractic activity. Then the entire chamber was incubated at 37°C for 6 h to initiate migration. Non-migrated cells were wiped off with a cotton swab and then the filter was fixed and stained with hematoxylin (Sigma) to define the cell nuclei. Chemotaxis was assessed by counting the number of migrated cells in five (at 400× magnification) random microscopy fields per well [20, 21]. All experiments were performed in triplicate. Chemokines-induced cell migration minus spontaneous migration in PBS served as control and was designated as 100% migration for each experiment.

### 2.7. Statistical Analysis

All values in the text and figures represent means ± SE. Data were analyzed by un-paired Student's *t*-test. *P *< .05 was considered statistically significant.

## 3. Results

### 3.1. ER and Three Quinazoline Alkaloids Suppressed H1N1-Induced RANTES and MCP-1 Production in Human Lung Epithelial Cells

**Figure 1:**
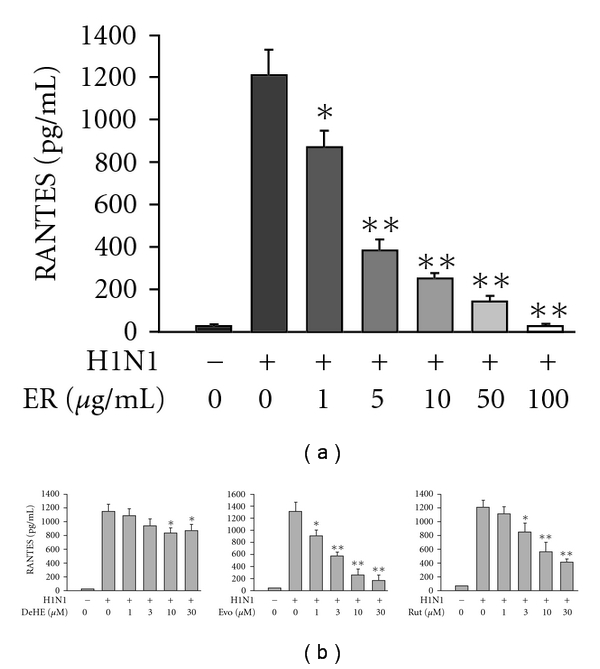
Effects of ER (a) and three major quinazoline alkaloids (b) on H1N1-stimulated RANTES production by A549 human bronchial epithelial cells. RANTES concentration was determined at 72 h after virus inoculation. Data reported are mean ± SE of four independent experiments, each performed in triplicate. *P < .05 and **P < .01 indicate significant differences as compared with H1N1 inoculation alone.

RANTES concentration was very low in the uninfected cell supernatant after a 72-h culture period. Infection of the A549 cells with influenza A/H1N1 evoked a seriously enhanced secretion of RANTES from basal (23 ± 9 pg ml^−1^)) to 1207 ± 114 pg ml^−1^ measured at 72 h. At the non-cytotoxic doses (1–100 *μ*g ml^−1^), ER exerted a consistent inhibitory responsiveness on H1N1-stimulated RANTES accumulation with an ID_50_ value of 1.9 ± 0.4 *μ*g ml^−1^ (Figure 1(a)). Three major components (DeHE, Evo and Rut) at concentrations ranging from 1 to 30 *μ*M, although to differing extents, all inhibited H1N1-induced RANTES production.  Figure 1(b) shows that Evo (1, 3, 10 and 30 *μ*M) treatment inhibited virus-evoked RANTES production in a concentration-dependent manner: statistical significance was observed beginning at 1 *μ*M (*P *< .05) and suppression approaching basal was achieved by 30 *μ*M Evo (IC_50_: 2.1 ± 0.7 *μ*M). Rut also dose-dependently inhibited virus-evoked RANTES release with an IC_50_ of 8.3 ± 1.9 *μ*M, although inhibition obtained by 30 *μ*M was not complete. DeHE, however, was the least potent of these drugs in reducing (around 20% of inhibition at 30 *μ*M) H1N1-induced RANTES production in A549 cells. The data of cell viability assessed by MTT assay showed that all tested agents at concentrations used did not express significant cytotoxicity.

**Figure 2:**
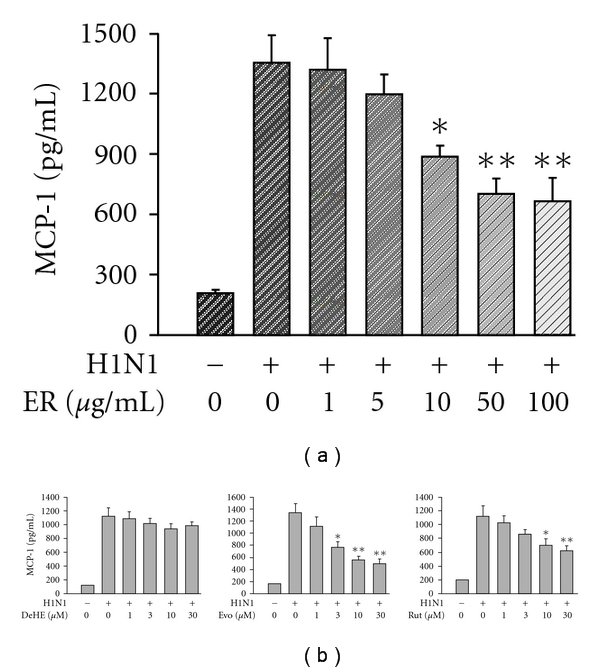
Effects of ER (a) and three major quinazoline alkaloids (b) on H1N1-stimulated MCP-1 production by A549 human bronchial epithelial cells. RANTES concentration was determined at 72 h after virus inoculation. Data reported are mean ± SE of five independent experiments, each performed in triplicate. *P < 0.05 and **P < .01 indicate significant differences as compared with H1N1 inoculation alone.

A significant increase in MCP-1 production was also noted after H1N1 infection.  Figure 2(a) shows that MCP-1 concentration was increased from basal (198 ± 21 pg ml^−1^) to 1357 ± 131 pg ml^−1^ at 72 h post-infection and such an induction could be suppressed by ER concentration dependently. As with RANTES production, Evo was the most potent in suppressing MCP-1 formation with an IC_50_ value of 8.7 ± 1.3 *μ*M (Figure 2(b)). In the case of Rut, a low dose (1 and 3 *μ*M) failed to, but a higher dose (10 and 30 *μ*M) significantly reduced H1N1-evoked MCP-1 secretion. The trend of inhibition was observed with DeHE, but not to a level of statistical significance.

### 3.2. ER and Three Quinazoline Alkaloids Reduced RANTES- and MCP-1-Induced Chemotactic Migration of Macrophages

**Figure 3:**
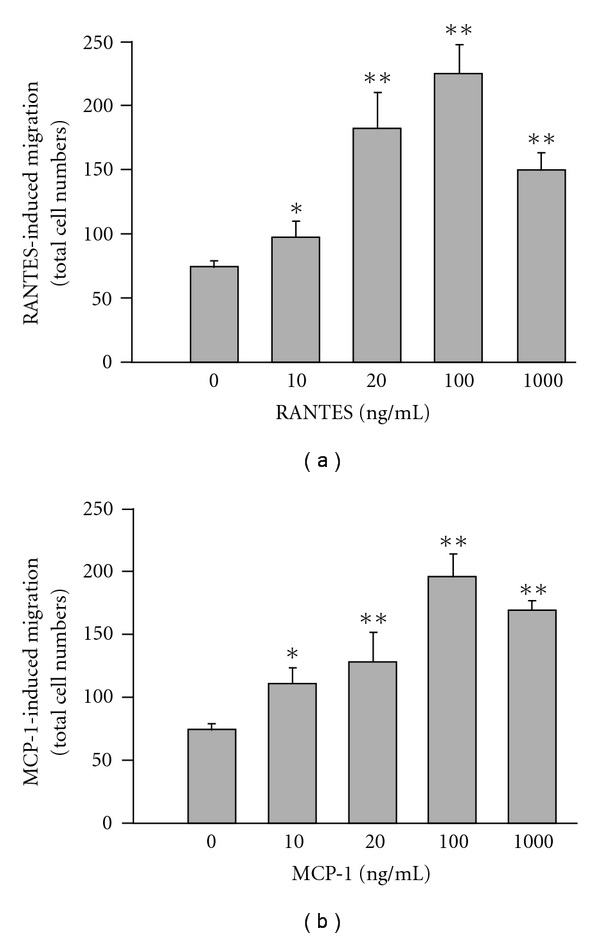
Concentration-related cell migration of HL-60-differentiated macrophages in response to RANTES (a) and MCP-1 (b). Cells were placed in the upper well and chemokines were added into the lower wells of the chamber for the assessment of migration activity. The entire chamber was then incubated for 6 h at 37°C. Migration was assessed by counting migrated cells in five microscopic fields per well at 400× magnification. Data reported are mean ± SE of six independent experiments, each performed in triplicate. *P < .05 and **P < .01 indicate degrees of statistical significance of difference as compared to un-stimulated cells.

**Figure 4:**
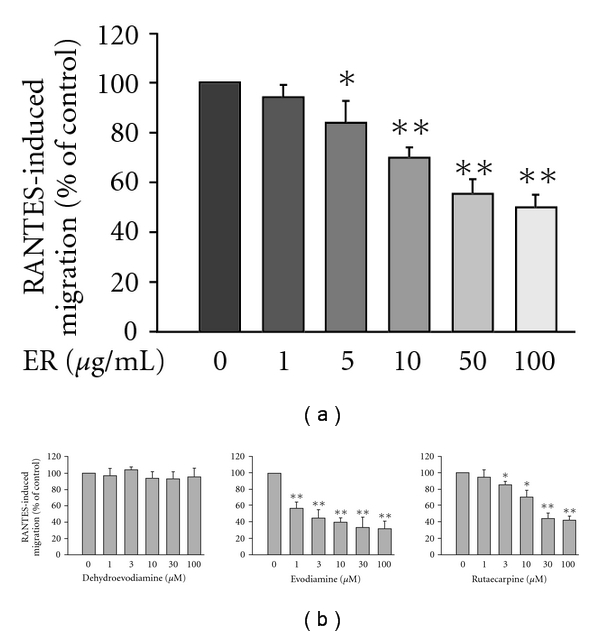
Effects of ER (a) and three major alkaloids (b) on RANTES-induced migration of HL-60-differentiated macrophages. Cells pre-incubated with respective tested agents for 30 min were plated into the upper wells of the chamber. RANTES (100 ng ml^−1^) was then added to the lower wells to induce cell migration for 6 h. Migration was assessed by counting migrated cells in five microscopic fields per well at 400× magnifications. RANTES-induced cell migration minus spontaneous migration in PBS served as control and was designated as 100%. Data reported are mean ± SE of six independent experiments, each performed in triplicate. *P < .05 and **P < .01 indicate degrees of statistical significance of difference as compared to samples without tested agents treatment.

Figure 3(a) shows that RANTES at concentrations ranging from 10 to 1000 ng ml^−1^ was able to evoke cell migration of HL-60-differentiated macrophages with a peak activity noted at 100 ng ml^−1^ (total migrated cell number of 223 ± 31). Time course analysis from a preliminary study also showed an increase in cell migration with time, reaching a maximum between 4 and 6 h. Therefore, 100 ng ml^−1^ of RANTES and a migration period of 6 h were selected for further experiments. The results showed that ER alone (1–100 *μ*g ml^−1^) did not influence spontaneous transmigration (data not shown), but concentration dependently inhibited RANTES-stimulated chemotactic migration with an IC_50_ value of 79.2 ± 8.4 *μ*g ml^−1^ (Figure 4(a)). None of the concentrations of ER used reduced cell viability (>95%), as assayed by MTT exclusion. Further, we studied the effect of three alkaloids on RANTES-induced migration of HL-60-differentiated macrophages. The result showed that Evo and Rut have the ability to inhibit RANTES-evoked cell migration in a concentration-dependent manner (Figure 4(b)). In contrast, DeHE failed to cause such a phenomenon.

**Figure 5:**
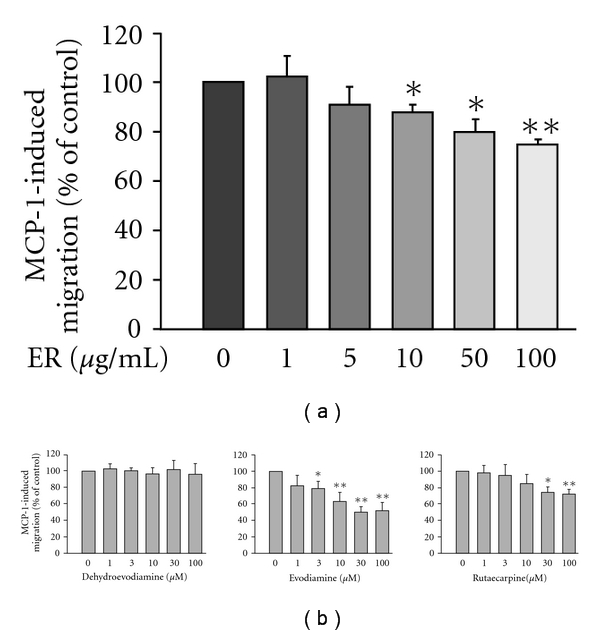
Effects of ER (a) and three major alkaloids (b) on MCP-1-induced migration of HL-60-differentiated macrophages. Cells pre-incubated with respective tested agents for 30 min were plated into the upper wells of the chamber. MCP-1 (100 ng ml^−1^) was then added to the lower wells to induce cell migration for 6 h. Migration was assessed by counting migrated cells in five microscopic fields per well at 400× magnifications. MCP-1-induced cell migration minus spontaneous migration in PBS served as control and was designated as 100%. Data reported are mean ± SE of six independent experiments, each performed in triplicate. *P < .05 and **P < .01 indicate degrees of statistical significance of difference as compared to samples without tested agents treatment.

Whether HL-60-differentiated macrophages could migrate toward MCP-1 and the effects of ER and its active components on this phenomenon were further studied. Compared with the unstimulated condition (displayed spontaneous migration with a total cell number of 68 ± 9), macrophages treated with MCP-1 evoked significantly chemotactic migration with peak activity occurring at 100 ng ml^−1^ (total migrated cell number of 194 ± 22), pointing out that MCP-1 is a chemotactic agent in inducing migration of HL-60-differentiated macrophages (Figure 3(b)). We next examined whether ER could affect the responsiveness of macrophages to MCP-1. We found that ER also suppressed MCP-1-evoked cell migration, although to a lesser extent than the suppressed RANTES response (Figure 5(a)). The IC_50_ value for ER to inhibit MCP-1-induced migration was 235.1 ± 11.6 *μ*g ml^−1^. Except for DeHE, there were trends for the others to inhibit MCP-1-evoked cell migration (Figure 5(b)). The IC_50_ levels for Evo and Rut to inhibit such a response were 67.2 ± 9.5 and 218.0 ± 25.4 *μ*M, respectively. Nevertheless, these trends were less effective than inhibiting migration toward RANTES.

## 4. Discussion

Influenza A virus attacks upper respiratory tract and replicates in epithelial cells resulting in the production of cytokines and chemokines that recruit leukocytes to infiltrate the infected tissues. Severe influenza A virus infection may cause complicated secondary bacterial infection and inflammatory response, lung infiltration and fibrosis, failure of respiration and even death. The new drug development strategy pointed to preventing macrophage invasion by interfering with chemokine secretion; chemokine receptors expression or cell migration may serve as potential agents to prevent deterioration and interrupt the pathogenesis following viral infection. After H1N1 infection, the alveolar epithelial cells can produce various kinds of chemokines to attract immune cell (such as monocyte/macrophage) [2–5]. RANTES and MCP-1 belonging to the CC chemokines family are the two most potent chemoattractants for monocyte/macrophage. In our preliminary study, we have found that after H1N1 inoculation, the production of these two chemokines by A549 cells was more pronounced than the other CC chemokinse such as MIP-1*α* and MIP-1*β* [19]. Furthermore, MCP-1 and RANTES could evoke more significant chemotactic migration than the other chemokines did when applying the recombinant chemokine proteins to the lower chamber of the transwell [19]. Therefore, MCP-1 and RANTES were chosen as two candidates to assess the effects of *E. rutaecarpa* and its bioactive components in the present study.

*Evodiae fructus* (known in Chinese as wu-chu-yu), is the dried, nearly ripe fruit of *E. rutaecarpa* (Juss.) Benth. (Rutaceae). It is officially listed in the Chinese Pharmacopoeia and is used as an analgesic, anti-emetic, astringent and anti-inflammatory agent and for treating hypertension. It is also used as a remedy for gastrointestinal disorders (abdominal pain and dysentery), headaches, amenorrhea and post-partum hemorrhage; and for treating bacterial infections (e.g, pneumonia bacteria) and inflammation-related disorders such as eczema and ulcerative stomatitis [22]. *Evodia rutaecarpa* had been used for treating infectious diseases because of the anti-inflammatory properties of its active components. Early in 1999, Moon et al. reported that Rut is a new class of COX-2 inhibitor [23]. We also found that DeHE and Evo could suppress LPS-induced NO production [8] and *E. rutaecarpa* was able to protect against circulation failure and organ dysfunction in endotoxaemic rats through modulating nitric oxide release [9]. Recently, Ko et al. found that the ER and its four bioactive components (including DeHE, Evo and Rut) all displayed anti-inflammatory activities, which could be partially explained by their differing potentials for inhibiting NADPH oxidase-dependent reactive oxygen species and/or iNOS-dependent NO production in activated inflammatory cells [14]. On the other hand, Liu et al. [24] reported that Evo repressed not only COX-2 and inducible nitric oxide synthase (iNOS) expression but also HIF-A accumulation in a concentration-dependent manner under hypoxic conditions [24]. Furthermore, Yarosh et al. [13] found ER extract was a potent inhibitor of UVB-induced pgE2 released by keratinocytes in culture. Twice daily application of 0.1%–1% ER extract for 2 weeks also significantly inhibited erythema following a methyl nicotinate (an NO inducer) challenge in human skin.

Here, we report that *E. rutaecarpa* and three major alkaloids (DeHE, Evo and Rut) isolated from this herb were able to inhibit pro-inflammatory chemokines production after H1N1 infection and subsequently chemotactic migration. We found that ER (the ethanol extract of *E. rutaecarpa*) had the ability to abrogate RANTES and MCP-1 production by H1N1-infected human alveolar epithelial cells (A549). Three major alkaloids also displayed different abilities to suppress chemokines secretion with the inhibitory order of Evo > Rut > DeHE, indicating that the coordination by Evo and Rut may significantly contribute to the chemokine inhibitory effect by ER. Direct toxicity of all tested compounds on A549 cells is an unlikely explanation for their inhibitory effects on chemokines production base without any clear cytotoxicity as measured by the MTT assay. In fact, we have investigated the effects of water extract and ER on H1N1-induced chemokines (RANTES and MCP-1) secretion in A549 alveolar epithelial cell as well as recombinant chemokine proteins-evoked migration of HL-60-differentiated macrophages in our preliminary study. Results showed that ER selectively attenuated H1N1 infection-evoked RANTRES/MCP-1 production and RANTRES/MCP-1 evoked cell migration, but the water extract of *E. rutaecarpa* failed to affect such responsiveness (data not shown). Unlike DeHE, Evo and Rut, the other two minor components isolated from ER named Q and S (Chiou et al., unpublished data), affected neither chemokines production nor chemotactic migration. Thus, we concluded that the effects of ER and its three major active components (DeHE, Evo and Rut) on chemokines production and cell migration were not due to non-specific false response.

During the influenza virus infection, viral RNA and viral proteins have been shown to activate the nuclear transcription factor NF-*κ*B resulting in many inflammatory genes transcription, including chemokines [25, 26]. Takada et al. and Choi et al. reported that Evo abolished constitutive and inducible NF-*κ*B activation by inhibiting I*κ*B kinase activation [11, 27]. Therefore, the inhibitory mechanisms of Evo on chemokines production may probably be attributed to interference with NF-*κ*B activation. Although they consistently reported that Rut did not inhibit NF-*κ*B activation, whether Rut reduced chemokines expression by affecting the other transcriptional factors activation remains to be further studied.

Macrophages play a key role in host defenses against invading micro-organisms. In response to pathogens, macrophages migrate along the chemokine gradient toward the infected tissue and finally engulf the pathogens by phagocytosis [28]. However, inappropriate recruitment and infiltration of the infected tissues may subsequently induce multiple sclerosis. Thus, drugs preventing macrophage invasion by interfering with chemokines secretion and subsequently evoking chemotactic migration may serve as potential agents to interrupt the inflammatory pathogenesis after severe viral infection. Here we report that ER (the ethanol extract of *E. rutaecarpa*) was able to inhibit RANTES-induced chemotactic migration of macrophages. Indeed, inhibition was not only observed with RANTES-induced migration. Our result indicated that ER also inhibited cell migration in response to MCP-1, although with less effectiveness (IC_50_: 79.2 ± 8.4 versus 235.1 ± 11.6 *μ*g ml^−1^). Data from the present study and our previous publications [21] all indicated that the dose response for chemokines-induced cell migration was bell shaped because more higher concentrations of chemokines did not result in further increase in migrated cell numbers. Such bell-shaped cell migration response was also observed in other studies [29, 30].

Because ER inhibited not only RANTES-induced cell migration but also MCP-1-induced cell migration, it may be postulated that ER may act as a broad inhibitor to abrogate inflammatory processes attributed to chemokine/cytokine dysregulation. To evaluate whether the anti-migration effect of ER was correlated with the three major bioactive components, their effects on both RANTES- and MCP-1-stimulated chemotactic migration in macrophages were also determined. Our results indicated that Evo was consistently the most potent agent among these components responsible for the ER's anti-migration effect and that Rut was accountable for such inhibition by ER too. It is reasonable that such activity contributed to the anti-migrating effect by Evo and Rut toward RANTES and MCP-1, respectively. We found that DeHE was the least potent drug throughout the experiment. Ogasawara et al. reported that Evo has remarkable inhibitory activity against hepatocyte growth factor (HGF)-induced migration of tumor cells (colon 26-L5 carcinoma, B16-F10 melanoma and Lewis lung carcinoma) [21–33]. Recently, Heo et al. examined the inhibitory effect of Evo and Rut on LIGHT-induced migration in human monocytes, THP-1. They found that Evo and Rut can inhibit LIGHT-induced migration by decreasing the expression of CCR1 and CCR2, two chemokine receptors that recognize RANTES and MCP-1, respectively [34]. We suggested the action site for DeHE, Evo and Rut may target inside the cell. To interrupt intracellular signal transductions mediating chemokine production, chemokine receptor expression or consequent cell migration, these compounds have to penetrate the cell membrane. According to the dissolved characteristic, Evo and Rut are lipophilic but DeHE is relatively more hydrophilic. This means DeHe has more difficultly penetrating the cell.

**Figure 6:**
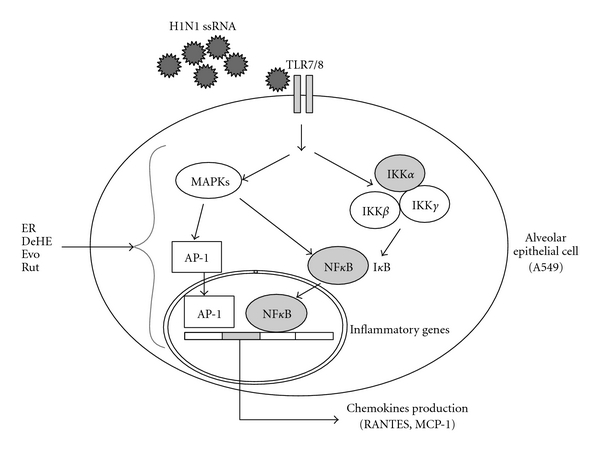
A schematic diagram of H1N1-induced signaling and inflammatory genes expression. Single-strand RNA (ssRNA) generated following H1N1 infection primarily activates cellular inflammatory genes via the Toll-like receptor 7/8 (TLR7/8)-dependent pathway to activate mitogen-activated protein kinases (MAPKs) and inhibitory *κ*B kinase complex (IKK*α*/*β*/*γ*), respectively. Both activating protein-1 (AP-1) and nuclear factor *κ*B (NF*κ*B) are activated as downstream events of TLR7/8-dependent activation of chemokines (including RANTES and MCP-1) production. An arrowhead proposed the possible multiple intracellular target sites for ER, DeHE, Evo and/or Rut to inhibit chemokines production in H1N1-infected A549 cells. (ER, the ethanol extract of *E. rutaecarpa*; DeHE, dehydroevodiamine; Evo, evodiamine; Rut, rutaecarpine).

**Figure 7:**
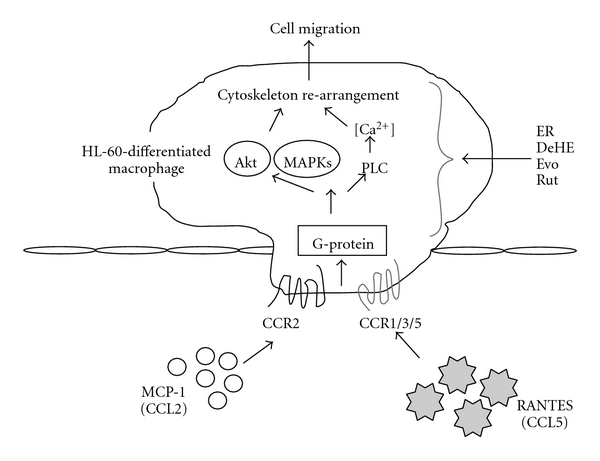
A schematic presentation of the potential signaling pathway triggered by RANTES and MCP-1 through CC chemokine receptor 1/3/5 (CCR1/3/5) and CCR2. RANTES (belong to CC chemokine ligand 5) and MCP-1 (belong to CC chemokine ligand 2) stimulated cell migration via a G-protein-dependent pathway. MAPKs, phospholipase C (PLC) and Akt are activated as downstream events of G-protein-dependent activation of cytoskeleton re-arrangement. An arrowhead proposed the possible multiple intracellular target sites for ER, DeHE, Evo and/or Rut to inhibit RANTES- or MCP-1-evoked cell migration in HL-60-differentiated macrophages. (ER, the ethanol extract of *E. rutaecarpa*; DeHE, dehydroevodiamine; Evo, evodiamine; Rut, rutaecarpine).

In conclusion, the anti-inflammatory properties of the ER could be due, at least in part, to the different potentials of its three major alkaloids (DeHE, Evo and Rut) in inhibiting chemokines production and/or chemotactic migration, although we did not examine the action mechanism about how DeHE, Evo and Rut repressed chemokines production and cell migration in detail. Based on our results and other's findings, we suggested that the possible action sites for these compounds may target inside the cell as showed in Figures 6 and 7, respectively. Our results indicated that *E. rutaecarpa* possesses therapeutic potential through its capacity to abrogate chemokines secretion and limit the infiltration of immune cells into infective sites. This may impede the progression and aggravation of inflammation given the migration of immune cells plays an important role in the outcome of lung infiltration and fibrosis. This is the first report offering the possibility of using this botanical extract or a biomimetic mixture of quinazoline alkaloids (especially Evo and Rut) to act synergistically to ameliorate chronic inflammatory conditions following H1N1 infection.
